# Comparison with first round findings of faecal haemoglobin concentrations and clinical outcomes in the second round of a biennial faecal immunochemical test based colorectal cancer screening programme

**DOI:** 10.1177/09691413221110012

**Published:** 2022-06-23

**Authors:** Gavin RC Clark, Callum G Fraser, Judith A Strachan, Robert JC Steele

**Affiliations:** 1 9571 Public Health Scotland, Edinburgh, UK; 2 Centre for Research into Cancer Prevention and Screening, University of Dundee, UK; 3 Blood Sciences and Scottish Bowel Screening Laboratory, Ninewells Hospital and Medical School, Dundee, UK

**Keywords:** Adenoma, colonoscopy, colorectal cancer screening, faecal immunochemical test, faecal haemoglobin

## Abstract

**Objective:**

How faecal haemoglobin concentrations (f-Hb) vary from one round to the next in a colorectal cancer (CRC) screening programme, and relate to colonoscopy findings, are unknown. Our aim was to use data from the first two rounds of the faecal immunochemical test (FIT) based Scottish Bowel Screening Programme (SBoSP) to explore these issues.

**Methods:**

Faecal haemoglobin concentration (f-Hb) percentiles in the second round were compared with those in the first when the first round yielded a negative FIT result (<80 µg Hb/g faeces), a positive FIT but no colonoscopy, CRC, all adenoma, and a negative colonoscopy. In addition, the outcomes in the first and second rounds were compared.

**Results:**

The profiles of f-Hb in the first and second rounds differed in (a) those who had had a negative FIT result in the first round and (b) those in whom neoplastic pathology had been found. In contrast, the pattern of difference between profiles in those who had had a negative colonoscopy was very similar to that in those in whom an adenoma had been found. In addition, the risk of CRC being diagnosed in the second round after a negative colonoscopy in the first was 3.0%, not very different to that after a negative test result (4.9%).

**Conclusions:**

Adenomas may be rarely the cause of a positive FIT result. An alternative explanation as to why these are detected using FIT is required. In addition, a negative colonoscopy for a positive FIT result does not rule out the finding of significant neoplastic pathology in the next round.

## Introduction

Randomised controlled trials (RCT) have shown that mortality from colorectal cancer (CRC) can be reduced by population screening using guaiac faecal occult blood tests (gFOBTs).^1^ However, gFOBTs have many disadvantages and have been almost completely superseded by quantitative faecal immunochemical tests (FITs) for haemoglobin in CRC screening since they are specific for human haemoglobin, do not need dietary or drug restriction, are easier to perform with hygienic specimen collection devices, usually only a single sample is required (and therefore associated with higher population uptake), and the generation of a result is automated, thus removing visual qualitative interpretation.^2,3^ In addition, a quantitative FIT provides an estimate of the faecal haemoglobin concentration (f-Hb) so that the threshold used to trigger an invitation to undergo colonoscopy can be adjusted to suit colonoscopy capacity.

Because most regional and national FIT-based CRC screening programmes are of recent onset, there is relatively little known about the relationship between the FIT results and clinical outcomes in one round of screening and the results and clinical outcomes in the subsequent round. We have shown that, when a cut-off threshold of 80 µg Hb/g faeces is used, the f-Hb in those with a “negative” FIT result (i.e. with f-Hb less than the threshold) is higher in those diagnosed with advanced neoplasia in the next round.^4^ It is also known that an increasing f-Hb in those with a negative FIT result, when a threshold of 20 µg Hb/g faeces is employed, is associated with an increasing risk of advanced neoplasia or interval cancer in subsequent screening rounds.^5–7^ In addition, data from a trial comparing gFOBT and FIT in The Netherlands demonstrated that, when the threshold for positivity used was 10 µg Hb/g faeces, although the positivity did not change after repeated CRC screening, the positive predictive values (PPV) of FIT for both CRC and advanced neoplasia were significantly lower in those second-round participants who had received a negative test result in the first round.^8^

However, to our knowledge, there has been no comparison between f-Hb in one round of FIT screening and f-Hb in the subsequent round, and how this varies with the clinical outcomes achieved in the first round. The Scottish Bowel Screening Programme (SBoSP), in the UK, re-invites those who underwent colonoscopy in the previous round, and we have therefore been able to use data from the first two rounds of the FIT-based SBoSP to examine f-Hb in those participants completing a FIT in both rounds in order to determine how this changes in the context of the findings at colonoscopy in the first round. In addition, we have investigated how the findings at colonoscopy in the first round compare with the findings at colonoscopy in the second round in those with a second “positive” FIT result (i.e. f-Hb equal to, or greater than, the applied threshold).

## Methods

Data from the first two rounds of FIT-based screening in the SBoSP were used for the analysis. The first round commenced in November 2017 and the second in November 2019. The SBoSP invites every man and woman registered with a general practitioner and aged between 50 and 74 years to participate by means of an invitation letter, a specimen collection device (HM-JACKarc, Minaris Medical Co., Ltd, Tokyo, Japan), instructions and an explanatory leaflet, all mailed to their place of abode. Participants over the age of 75 years can opt-in by contacting the programme. All accepting the invitation complete the faecal collection themselves using the device, which is then mailed back in a special envelope to the Scottish Bowel Screening Laboratory in Dundee for analysis. A “positive” FIT result is defined as f-Hb ≥80 µg Hb/g faeces, and this is communicated to the relevant territorial National Health Service (NHS) Board so that further investigation (usually colonoscopy) and treatment, if required, can be provided. The NHS Boards are required to upload screening endoscopy and pathology data on a regular basis to Public Health Scotland.

### Faecal haemoglobin percentiles in round 1 and round 2

Participants with FIT results in both their first and second round were identified using their unique Community Health Index (CHI) number. Since f-Hb in screening populations does not follow a normal or log-normal distribution, non-parametric methods of statistical analysis were used; to investigate the f-Hb profiles, data were derived for the 25^th^, 50^th^, 75^th^, 90^th^, 95^th^ and 97.5^th^ percentiles. Use of data outside the analytical measurement range, which is 7–400 μg Hb/g faeces for the FIT system used in this study, has become usual for research as well as clinical purposes at low f-Hb concentrations below the limit of detection, and an analogous strategy has been adopted here through examination of all results ≥80 μg Hb/g faeces, including those greater than the upper measurement limit of 400 μg Hb/g faeces. The significance of differences between the first and second round distributions was assessed using the Wilcoxon signed-rank test

Within the cohort, further sub-cohorts were derived based on their first-round outcome – negative FIT result, positive FIT with no colonoscopy, all adenoma as most serious outcome, CRC, and negative colonoscopy (i.e. no neoplasia found). Those with adenoma in their first round were also separated into low risk and higher-risk adenoma (LRA and HRA, respectively). LRA was defined as less than three adenomas, all less than 10 mm, and HRA as at least one adenoma of 10 mm or greater, or three or more adenomas in the same participant, as recommended by the 2001 British Society of Gastroenterology guidelines on adenoma surveillance.^9^ These have been recently updated,^10^ but the 2001 guidance has been used in Scotland since the start of the SBoSP and the new guidelines were not yet published when the data for this study were generated. The data are reported electronically by the NHS Boards responsible for regional health care, and the IT system and approaches have been retained to facilitate data analysis over time using consistent terminology and classification.

### Outcomes in the second round

The outcomes in the second round were also examined, specifically second round positivity, uptake of subsequent colonoscopy, and positive predictive value (PPV) for all adenoma, LRA, HRA, and CRC in those who underwent a second round colonoscopy. 95% confidence intervals were calculated for each outcome. All analyses were performed using RStudio version 3.6.1. Formal ethical approval was not required because individual participants were not approached, only routinely collected data were utilised, and all data were anonymised. Patients and/or the public were not involved in the design, conduct or reporting or dissemination plans of this research.

## Results

### Faecal haemoglobin percentiles in round 1 and round 2

**Figure 1.**
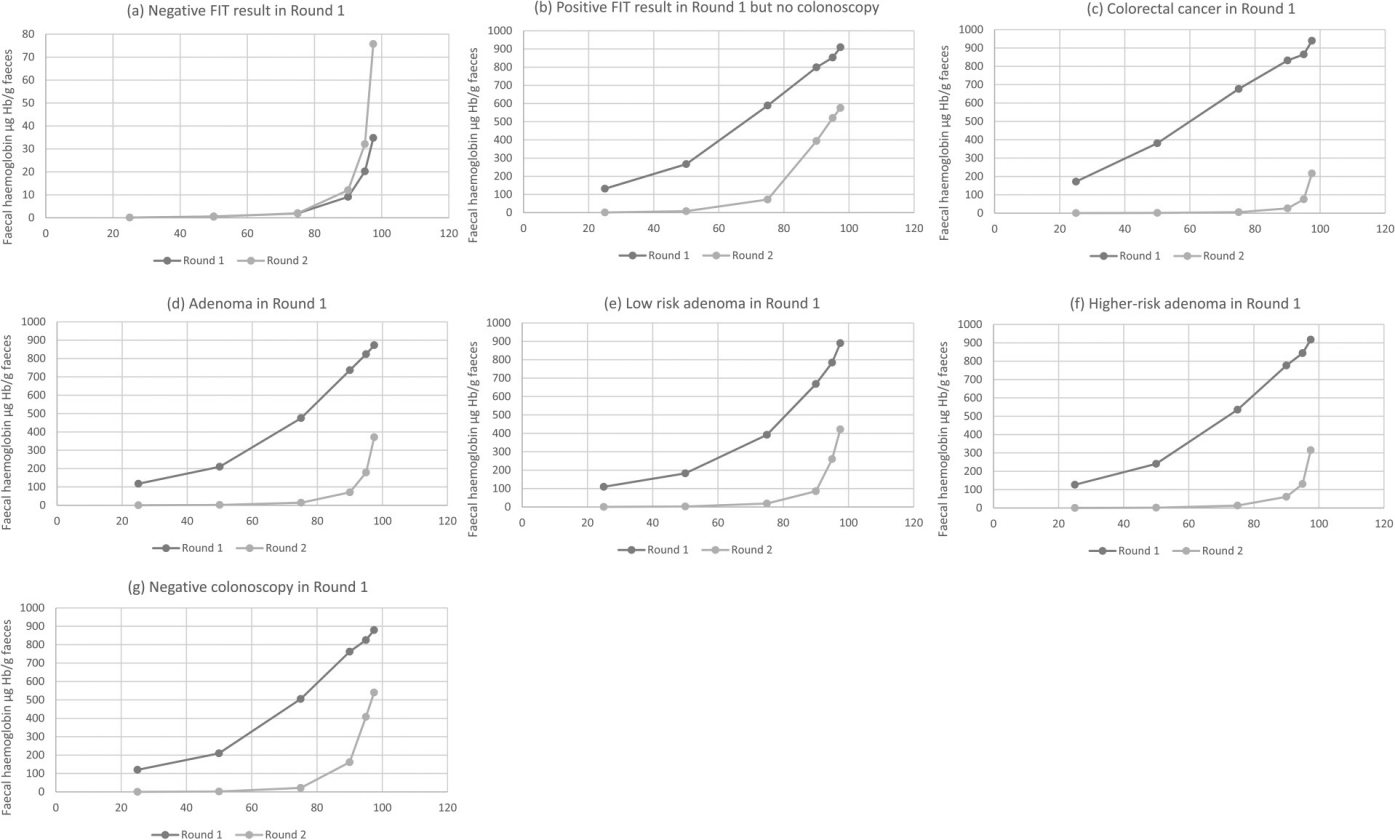
Faecal haemoglobin concentration (f-Hb) percentiles in the first and second rounds for participants who were found to have in round 1: (a) negative faecal immunochemical test (FIT) result, (b) positive FIT result but no colonoscopy, (c) colorectal cancer, (d) adenoma, (e) low risk adenoma, (f) higher-risk adenoma, and (g) negative colonoscopy.

 Figure 1(a) shows the f-Hb percentiles in those with a “negative” FIT result (f-Hb <80 µg Hb/g faeces) in the first round compared with the same participants in the second round; on visual inspection, the f-Hb profile in the second round is slightly higher than in the first Figure 1(b) shows the percentiles for those with a positive FIT result (f-Hb ≥80 µg Hb/g faeces) and no colonoscopy in the first round compared with the same participants in the second round and, in this instance, the f-Hb distribution profile in the second round is lower than that in the first Figure 1(c) shows the percentiles for those with a positive FIT result and a diagnosis of CRC in the first round again compared with the same participants in the second round and, here, the f-Hb distribution profile in the second round is markedly lower than that in the first Figure 1(d)-1(f) show similar data for those with adenoma, LRA and HRA diagnosed in the first round. Figure 1(g) shows the percentiles for those who had a positive FIT result and a “negative” colonoscopy (no neoplastic pathology) in the first round compared with the same participants in the second round. Interestingly, the profiles for those with any finding of adenoma (but especially LRA) in the first round were almost identical to those for whom the first round colonoscopy had been negative. The difference between second and first round f-Hb percentiles was found to be highly statistically significant, for all cohorts, at the p < 0.001 level. The numerical data for the f-Hb percentiles for all of the groups are documented in Supplementary Table 1.

### Outcomes in the second round

 Table 1 shows the second round outcomes of those with a positive FIT result in each of the first round categories (negative FIT result, positive FIT result but no colonoscopy, all adenoma, LRA, HRA, CRC, negative colonoscopy) expressed as positivity and positive predictive value (PPV) (in those who went on to have colonoscopy) for all adenoma, LRA, HRA and CRC.

**Table 1.** Second round outcomes of those with a positive FIT result in each of the first round categories (negative FIT result, positive FIT result but no colonoscopy, colorectal cancer, all adenoma, low risk adenoma, higher-risk adenoma and negative colonoscopy), expressed as positivity (%) and PPV % (with 95% CI) in those who went on to have colonoscopy. .

	Second round outcomes							
First round outcomes	N	Positive FIT* result (n)	Positive FIT result % (95% CI)	Positive FIT result with colonoscopy performed % (95% CI)	All adenoma: PPV % (95% CI)	Low risk adenoma: PPV % (95% CI)	Higher risk adenoma: PPV % (95% CI)	Colorectal cancer: PPV % (95% CI)
Negative FIT result	192,347	4573	2.4 (2.3−2.4)	74.8 (73.5−76.0)	45.9 (44.2−47.6)	18.6 (17.4−20.0)	25.7 (24.3−27.2)	4.9 (4.2−5.7)
Positive FIT result but no colonoscopy	838	201	24.0 (21.2−27.0)	32.8 (26.7−39.6)	33.3 (23.1−45.3)	9.1 (4.2−18.4)	22.7 (14.3−34.2)	4.5 (1.6−12.5)
Colorectal cancer	223	11	4.9 (2.8−8.6)	45.5 (21.3−72.0)	40.0 (11.8−76.9)	0.0 (0.0−43.4)	40.0 (11.8−76.9)	0.0 (0.0−43.4)
All adenoma	2000	171	8.6 (7.4−9.9)	76.0 (69.1−81.8)	61.5 (53.0−69.5)	32.3 (24.9−40.8)	25.4 (18.7−33.5)	2.3 (0.7−6.6)
Low risk adenoma	876	92	10.5 (8.6−12.7)	78.3 (68.8−85.5)	62.5 (51.0−72.8)	38.9 (28.5−50.4)	19.4 (12.0−30.0)	2.8 (1.0−9.6)
Higher-risk adenoma	1103	76	6.9 (5.5−8.5)	75.0 (64.2−83.4)	59.6 (46.7−71.4)	22.8 (13.8−35.2)	33.3 (22.5−46.2)	1.8 (0.3−9.2)
Negative colonoscopy	2200	307	14.0 (12.6−15.5)	65.8 (60.3−70.9)	25.2 (19.8−31.7)	12.4 (8.5−17.6)	11.4 (7.7−16.5)	3.0 (1.4−6.3)

FIT: faecal immunochemical test for haemoglobin; PPV: positive predictive value.

## Discussion

### Faecal haemoglobin percentiles in round 1 and round 2

The data presented here provide a detailed insight into how f-Hb varies from the first round to the second in a FIT-based CRC screening programme. Unsurprisingly, in those with a negative FIT result in the first round (i.e. <80 µg Hb/g faeces) the f-Hb profile shows a slight shift to higher f-Hb in the second round. This can be explained by increasing age (since all participants studied here were two years older, and f-Hb is known to increase with age^11,12^) and a consequent increased chance of developing neoplasia. Similarly, it might have been expected that in those with CRC diagnosed in the first round the profile of f-Hb would be markedly lower in the second round since, for the majority of participants, the bleeding lesion would have been removed early in the interval between the two screening episodes. Those with adenoma (both LRA and HRA) in the first round also experienced a shift to lower f-Hb in the profile, but considerably less than for CRC, especially those with LRA. It is also worth noting that those with a positive first round FIT result who did not undergo colonoscopy had a lower f-Hb profile in the second round but higher than in any of the other categories; this can be explained by a larger proportion of those in this category continuing to harbour neoplastic pathology into the second round than those in whom neoplasia had been detected and removed in the first round. This is borne out by the high PPV for CRC in the second round (4.5%) in this group (Table 1).

It is perhaps more surprising that the f-Hb profile in those who had a negative colonoscopy in the first round shifts to lower f-Hb in the second round, but this may be explained by the fact that an episode of bleeding from a benign, self-limiting condition, such as haemorrhoids or acute diverticulitis, is unlikely to occur again in the second round at the exact time of specimen collection. What is particularly interesting, however, is the observation that the pattern of difference in f-Hb profiles in those with adenoma, and particularly those with LRA, is very similar to that seen in those who had a negative colonoscopy. This suggests that participants with LRA are no more likely to have occult gastrointestinal bleeding than those in whom colonoscopy shows no obvious source for the blood, at least as reflected in the initial positive FIT result.

One implication for this finding is that the prevalence of adenoma in a FIT positive group, at least at the f-Hb threshold of ≥80 µg Hb/g faeces, is little different from an age and sex matched population at large. However, it is known that the prevalence of adenoma is higher in a population undergoing colonoscopy for a positive FIT result than in a similar population undergoing primary screening colonoscopy. Perhaps the best evidence for this comes from the recent SCREESCO trial from Sweden,^13^ in which 278,586 60-year-olds were randomised in a 1:2:6 ratio to colonoscopy, FIT at a threshold of ≥20 µg Hb/g faeces, or no screening. The risk of adenoma in those undergoing colonoscopy for a positive FIT result was significantly higher than in those undergoing first line screening colonoscopy; namely, 19.7% versus 8.7% for advanced adenoma (similar to our HRA) and 21.4% versus 17.3% for non-advanced adenoma (similar to our LRA).

Thus, a germane question is why should adenoma be more common in individuals with positive FIT results, when the risk, by implication from the change in f-Hb profile from one round of screening to a second, is similar to that in those with a negative colonoscopy after a positive FIT result? One explanation is that the adenomas themselves are rarely the direct source of the blood detected by FIT, but rather that occult colonic bleeding is a marker for increased risk of neoplasia. We have demonstrated that the presence of occult blood in faeces is associated with an increased risk of death from a number of causes all associated with a chronic inflammatory state.^14^ Moreover, we have recently reviewed the now substantial evidence that supports our hypothesis that the presence of occult blood in faeces (whether detected by gFOBT or FIT) is a marker of systemic inflammation manifested by low-grade colonic inflammation.^15^ This has been supported in a recent study on a randomly selected population of 20,694 participants followed in Denmark for 33 years in which an association between positive gFOBT, cause of death and mortality was observed; it was concluded that the presence of f-Hb might indicate the presence of systemic disease.^16^ Since most solid tumours arise against a background of chronic inflammation,^17^ a positive FIT result may indicate an increased *risk of adenoma* rather than *bleeding from an adenoma*.

### Outcomes in the second round

The second round outcomes (see Table 1) are also of considerable interest As might be expected, the FIT result positivity in the second round is lowest in those with a previous negative FIT result. The lowest rate of colonoscopy for a positive FIT result in the second round was in those who had not undergone colonoscopy in the first round, which can be explained by continued reluctance to be investigated or their continuance of lack of fitness for colonoscopy. The next lowest was in those in whom CRC had been diagnosed in the previous round, presumably because most of those found to have CRC would have been enrolled into surveillance colonoscopy programmes. The third lowest rate was in those with a previous negative colonoscopy, and this might be expected owing to a reluctance to undergo another unproductive and unpleasant procedure with some, albeit small, risk.^18^

The number of participants with CRC in the first round who underwent colonoscopy for a positive second round FIT was very small (n = 5) so only limited conclusions can be drawn, although it is interesting that 40.0% had HRA. A diagnosis of adenoma was also common in all those who had adenoma diagnosed in the first round, and the risk of a diagnosis of CRC was not negligible (2.3% overall). The highest risk of CRC was found in participants whose FIT result had been negative on the first round (4.9%), as might be expected given that very few would have benefitted from colonoscopy within the preceding two years. However, it is very interesting that a high risk of CRC remained in those who had undergone a colonoscopy in the first round that was negative (3.0%), and this group also had a substantial risk of adenoma (25.6%); this underlines the importance of continuing to offer screening to these individuals. This is in keeping with our previous work that found a 3.8% risk of CRC in the second round of a gFOBT programme among those with a negative colonoscopy in the first round,^19^ and with a study from the Taiwanese screening programme showing that amongst participants who had a negative colonoscopy those who underwent subsequent FIT were at lower risk of developing CRC than those who did not.^20^ The CRC cases in both of our studies, as well as many in the Taiwanese study, would be considered to be post-colonoscopy colorectal cancers,^21^ and the existence of these underlines the importance of continued screening.

## Conclusions

In summary, the data presented here illustrate the shift in profiles of f-Hb from one FIT screening round to a second, and suggest that removal of adenoma has little effect, probably owing to the mechanism whereby a positive FIT result predicts a future diagnosis of adenoma. The data also support the contention that those with a negative colonoscopy after a positive FIT result should not be denied the offer of screening at least at the subsequent round.

## Supplemental Material

sj-docx-1-msc-10.1177_09691413221110012 - Supplemental material for Comparison with first round findings of faecal haemoglobin concentrations and clinical outcomes in the second round of a biennial faecal immunochemical test based colorectal cancer screening programme Click here for additional data file. Supplemental material, sj-docx-1-msc-10.1177_09691413221110012 for Comparison with first round findings of faecal haemoglobin concentrations and clinical outcomes in the second round of a biennial faecal immunochemical test based colorectal cancer screening programme by Gavin RC Clark, Callum G Fraser and Judith A Strachan, Robert JC Steele in Journal of Medical Screening
