# Supplementary material for: Comparison with first round findings of faecal haemoglobin concentrations and clinical outcomes in the second round of a biennial faecal immunochemical test based colorectal cancer screening programme
Source: J Med Screen. 2022 Jun 23;29(4):249–54. doi: 10.1177/09691413221110012 (PMC9574424; doi:10.1177/09691413221110012)
Supplement: sj-docx-1-msc-10.1177_09691413221110012 - Supplemental material for Comparison with first round findings of faecal haemoglobin concentrations and clinical outcomes in the second round of a biennial faecal immunochemical test based colorectal cancer screening programme [file sj-docx-1-msc-10.1177_09691413221110012.docx]

**Supplementary Table 1.**

Faecal haemoglobin concentrations (µg Hb/g faeces) at 25th, 50th, 75th, 90th, 95th and 97.5th percentiles in the first and second rounds for participants in the first round who had: (a) negative faecal immunochemical test (FIT) result, (b) positive FIT result but no colonoscopy, (c) colorectal cancer, (d) all adenoma, (e) low risk adenoma, (f) higher risk adenoma, (g) negative colonoscopy.

| **Faecal haemoglobin concentration**  **(µg Hb/g faeces)** | | | | | | |
| --- | --- | --- | --- | --- | --- | --- |
| Percentile | 25th | 50th | 75th | 90th | 95th | 97.5th |
| (a) Faecal haemoglobin (µg Hb/g faeces) before a negative FIT result found in Round 1: Round 1 | 0 | 1 | 2 | 9 | 20 | 35 |
| (a) Faecal haemoglobin (µg Hb/g faeces) after a negative FIT result found in Round 1: Round 2 | 0 | 0 | 2 | 12 | 32 | 76 |
| (b) Faecal haemoglobin (µg Hb/g faeces) before a positive FIT result found but no colonoscopy in Round 1: Round 1 | 132 | 267 | 589 | 799 | 853 | 910 |
| (b) Faecal haemoglobin (µg Hb/g faeces) after a positive FIT result found but no colonoscopy in Round 1: Round 2 | 1 | 8 | 72 | 394 | 520 | 576 |
| (c) Faecal haemoglobin (µg Hb/g faeces) before a colorectal cancer found in Round 1: Round 1 | 171 | 381 | 677 | 832 | 865 | 939 |
| (c) Faecal haemoglobin (µg Hb/g faeces) after a colorectal cancer found in Round 1: Round 2 | 0 | 1 | 5 | 26 | 75 | 216 |
| (d) Faecal haemoglobin (µg Hb/g faeces) before an adenoma found in Round 1: Round 1 | 117 | 210 | 475 | 736 | 824 | 873 |
| (d) Faecal haemoglobin (µg Hb/g faeces) after an adenoma found in Round 1: Round 2 | 0 | 2 | 14 | 71 | 179 | 371 |
| (e) Faecal haemoglobin (µg Hb/g faeces) before a low risk adenoma found in Round 1: Round 1 | 109 | 183 | 392 | 668 | 784 | 890 |
| (e) Faecal haemoglobin (µg Hb/g faeces) after a low risk adenoma found in Round 1: Round 2 | 0 | 3 | 18 | 85 | 260 | 422 |
| (f) Faecal haemoglobin (µg Hb/g faeces) before a higher risk adenoma found in Round 1: Round 1 | 127 | 241 | 536 | 777 | 844 | 917 |
| (f) Faecal haemoglobin (µg Hb/g faeces) after a higher risk adenoma found in Round 1: Round 2 | 0 | 2 | 13 | 61 | 131 | 315 |
| (g) Faecal haemoglobin (µg Hb/g faeces) before a negative colonoscopy found in Round 1: Round 1 | 120 | 209 | 505 | 762 | 825 | 879 |
| (g) Faecal haemoglobin (µg Hb/g faeces) after a negative colonoscopy found in Round 1: Round 2 | 0 | 2 | 22 | 161 | 408 | 540 |
